# Serum anti-β2 spectrin IgA antibodies are produced in a CD4 T cell-independent manner in IgA nephropathy model mouse

**DOI:** 10.3389/fimmu.2026.1696625

**Published:** 2026-04-15

**Authors:** Hiroyuki Iwasaki, Yoshihito Nihei, Hitoshi Suzuki, Daisuke Kitamura, Yusuke Suzuki

**Affiliations:** 1 Department of Nephrology, Juntendo University Faculty of Medicine, Tokyo, Japan; 2 Division of Cancer Cell Biology, Research Institute for Biomedical Sciences (RIBS), Tokyo University of Science, Chiba, Japan; 3 Department of Nephrology, Juntendo University Urayasu Hospital, Chiba, Japan

**Keywords:** anti-β2 spectrin IgA antibody, autoantibody, CD4+ T cells, IgA nephropathy, IgA plasma cells

## Abstract

**Introduction:**

IgA nephropathy (IgAN) is characterized by deposition of IgA antibodies (Abs) in the glomerular mesangium. In IgAN, the mechanism by which IgA Abs are selectively deposited in the glomerular mesangial region remain unclear. Current study reported the presence of autoantibodies-IgA against β2 spectrin of mesangial cells in sera of IgAN model mice (gddY) and patients with IgAN, and identified β2-spectrin as the target antigen. And it found that IgA^+^ plasmablasts (PBs) secreting anti-β2-spectrin IgA Abs accumulated in the kidneys of gddY mice. These PBs exhibited a substantial accumulation of somatic hypermutations in both their heavy- and light-chain variable region genes.

**Methods:**

Lymphocytes from kidneys, renal lymph node, spleen, bone marrow and nasopharyngeal associated lymphoid tissue of 3-week-old ddY mice were analyzed by flow cytometry. To deplete CD4^+^ T cells in gddY mice, anti-CD4 monoclonal Abs (mAbs) were administered from 3 weeks of age. Renal phenotype (blood urea nitrogen (BUN), urinary albumin and renal pathology), the cell numbers of IgA^+^ PBs in the kidney and serum anti-β2 spectrin Abs were evaluated by Fuji-DRY-CHEM 7000V, enzyme-linked immunosorbent assay, microscopy, flow cytometry and western blotting, respectively.

**Results:**

Depletion of CD4^+^ cells in gddY mice caused significant reduction of the number of IgA^+^ PBs spontaneously accumulating in the kidneys in the anti-CD4 treatment (29.0 ± 12.1/10^5^ cells) than the control (154.0 ± 55.0/10^5^ cells). However, The results showed the level of urinary albumin and BUN were no difference in in the anti-CD4 treatment (5627.0 ± 1316.2 μg/dL, 40.3 ± 4.5 mg/dL, respectively) compared to the control (4766.5 ± 809.2 μg/dL, 69.9 ± 33.2, respectively). And also the treatment did not affect the production of anti-β2-spectrin IgA Abs in sera, glomerular IgA deposition nor pathology.

**Discussion:**

Our data suggest that the serum anti-β2-spectrin IgA Abs are produced in a CD4^+^ T cell-independent manner and the PBs infiltrating in the kidney are not the major source of serum anti-β2-spectrin IgA Abs at least in animal model. The present study will help to elucidate the mechanism of production of anti-β2-spectrin IgA Abs.

## 1 Introduction

IgAN is the most common type of primary glomerulonephritis in the world and characterized by deposition of IgA antibodies (Abs) in the glomerular mesangium (1). About 30-40% of patients with IgAN will progress to end stage renal disease in 10–20 years after diagnosis (2). In patients with IgAN, increases in auto-Abs such as ANA and ANCA have been reported (3). IgAN is considered to be an autoimmune disease characterized by the formation of IgA1-containing immune complexes in the circulation and glomerular depositions (4). However, the pathogenesis of IgAN remains poorly understood, thus, there are no specific treatment strategies in patients with IgAN (5–7). Marked ethnic and regional differences in the prevalence of IgAN suggest strong genetic and environmental influences, making the disease particularly challenging to interpret (8).

Currently, the multi-hit hypothesis has been proposed in pathogenesis of IgAN. Patients with IgAN often have an increase in circulating levels of IgA1 with galactose-deficient O-glycans in the hinge-region (Gd-IgA1) (Hit 1). Antibodies directed against Gd-IgA1 are synthesized and bind to Gd-IgA1 to form immune complexes (ICs) that accumulate in the glomerular mesangium (Hits 2 and 3). Because the clearance of these ICs in the liver decreases and their filtration in the glomerulus increases, ICs are deposited on mesangial region and activate mesangial cells, inducing their proliferation and secretion of extracellular matrix, cytokines, and chemokines, which results in renal injury (Hit 4) (5–7).

However, there are several aspects that cannot be explained by the multi-hit hypothesis: for instance, Gd-IgA1 is also increased in sera of a part of healthy relatives of IgAN patients (9) and IgA is deposited specifically in the glomerular mesangial region although the mere ICs would likely be deposited at various regions in the glomerulus. Thus, the mechanism by which IgA Abs are selectively deposited in the glomerular mesangial region in IgAN has not been elucidated.

To solve this problem, we have been studying the IgAN pathogenesis using an IgAN animal model termed gddY mice (10–12). GddY mice were isolated through intercrossing an early onset group of ddY mice, an outbred strain that has been studied as a model of spontaneous IgAN despite with variability in their disease onset. In contrast, all individual gddY mice exhibit proteinuria and glomerular IgA deposition by 8 weeks of age, followed by obvious renal failure and the pathology being similar to human IgAN (10–12).

We have identified IgA class auto-Abs against mesangial antigens, β2-spectrin, in the sera of gddY mice and IgAN patients (13). We also found that a significant number of IgA^+^ PBs accumulated in the kidneys of gddY mice and patients with IgAN, in line with accumulating evidence that plasma cells (PCs) secreting auto-Abs are present in inflamed tissues in several autoimmune diseases (14–17). In addition, these PBs in gddY mouse kidney produced IgA Auto-Abs that bind to β2-spectrin expressed on the surface of mesangial cells. Most of the variable region genes for IgA heavy and light chains cloned from single IgA^+^ PB isolated from the kidney of gddY mice contained substantial numbers of somatic hypermutations (13). Based on these discoveries, we hypothesized that the PBs in the kidney are generated in CD4^+^ T-cell dependent manner through the germinal center and could be a source of serum anti-β2-spectrin IgA Abs (18).

## 2 Materials and methods

All assay kits used in this study, including their manufacturers and lot numbers, are detailed. Experiments were conducted in a certified laboratory under biosafety level [BSL-1] conditions. All procedures involving potentially hazardous materials were performed following ethical guidelines and approved by the appropriate institutional review board.

**Figure 1:**
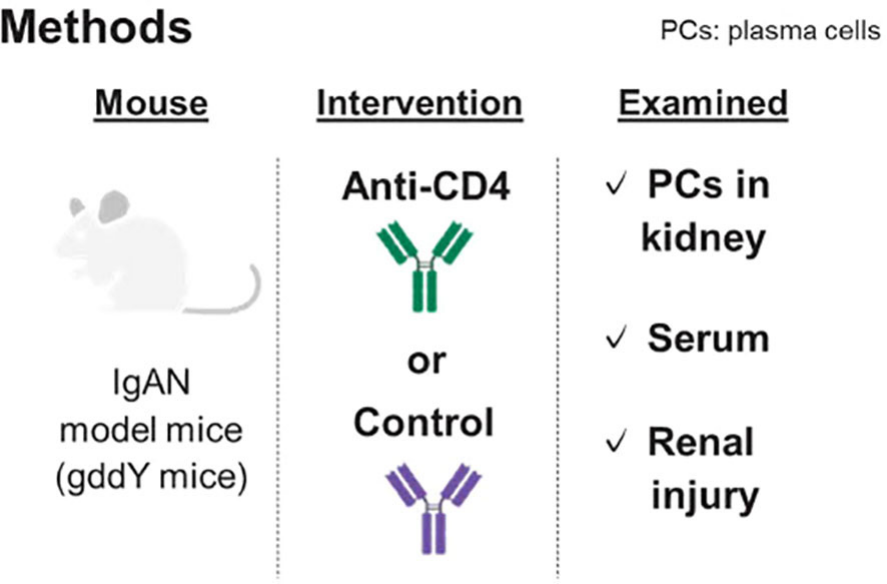
Overview of the study design. GddY mice were administered either anti-CD4 Abs or isotype-matched Abs, and kidney-infiltrating IgA^+^ plasma cells, serum Auto-Abs levels, and renal injury were evaluated. Created with BioRender.com.

A schematic representation of the study design is provided in **Figure 1**.

### 2.1 Mice

The gddY mice were generated by selectively mating individuals within an early-onset group of ddY mice for more than 20 generations (10, 11). All mice were maintained in Tokyo University of Science (TUS) mouse facility under specific pathogen-free conditions. Male mice are used in this study. Mouse procedures were performed under protocols approved by the TUS Animal Care and Use Committee. Mice were euthanized with CO2. The CO_2_ concentration increased gradually, replacing 30-70% of the chamber volume per minute. Infusion of CO_2_ continued for at least one minute after respiratory arrest. To deplete CD4^+^ T cells, 3-wk-old mice were injected intraperitoneally with 150 μg (day 0) and 250 μg (day 7, 14, and 21) of anti-mouse CD4 monoclonal Ab (mAb) (GK1.5) or isotype-matched mAb (LTF-2) (Bio X Cell), as described previously (19).

### 2.2 Immunofluorescence and optical microscopy

Immunofluorescence microscopy (IFM) was performed as previously reported (13). Briefly, freshly isolated mouse kidneys were embedded in OCT compound (Sakura Finetek), frozen in liquid nitrogen, and preserved at -80 °C until use. 6-μm-thick frozen sections were incubated in 3% BSA/PBS for 60 min at room temperature to block nonspecific staining, washed, and stained overnight at 4 °C with Abs. After washing, slides were mounted with ProLong Gold antifade reagent with DAPI (Invitrogen). To detect IgA, anti-mouse IgA-PE Ab (Abcam) was used. Renal tissue specimens for microscopic examination were fixed in 20% formaldehyde, embedded in paraffin, cut into 3-mm-thick sections, and then stained with periodic acid-Schiff (PAS). All the samples were examined with a BZ-x800 microscope (Keyence).

### 2.3 Tissue preparation and flow cytometry

Perfused kidneys were cut into 2- to 3-mm pieces and mononuclear cells in the kidney were isolated by Percoll gradient centrifugation after digestion with 0.6 mg/mL collagenase D (Roche) and 100 μg/mL DNase I (Roche) as described previously (13). Single-cell suspensions of kidney, renal lymph node (rLN), spleen, bone marrow (BM) and nasopharyngeal associated lymphoid tissue (NALT) were deprived of RBCs, then treated with FcγR-blocking mAb (2.4G2), and then stained with Abs against the following antigens; BD Biosciences: B220 (APC-Cy7), CD138 (BV421), IgA (biotin), CD4 (FITC, PE, APC or APC-Cy7), TCRβ (PE). Biotinated Abs were detected with streptavidin (VB421 or APC). For staining of IgA in PBs, intracellular staining was performed using Fixation and Permeabilization solution kit (BD Biosciences). All samples were analyzed using an FACS Calibur, or FACS Canto II (BD Biosciences). The data were analyzed using FlowJo (Tree Star).

### 2.4 Western blotting

Western blotting was performed as previously reported (20). FLAG-tagged recombinant full length of β2-spectrin protein were mixed with SDS sample buffer, boiled and used for SDS-PAGE, followed by immunoblotting using indicated sera or Abs. For use as the primary Abs, mouse sera were diluted at 1:25. Anti-mouse IgA Ab conjugated with HRP (1:8000) was used as the secondary Ab.

### 2.5 Recombinant protein production

Recombinant protein was generated as previously reported (13). FLAG-tagged mouse Sptbn1 were transiently expressed in HEK293T cells. From the lysates of the cells lysed with 1% NP-40, FLAG-tagged protein was purified using anti-FLAG M2 Affinity Gel (Sigma-Aldrich), according to the manufacturer’s instructions. The protein was eluted using 0.1 M glycine HCl (pH3.0) and neutralized immediately with 2 M Trizma base (Sigma-Aldrich). Purification was confirmed by Coomassie Brilliant Blue staining.

### 2.6 Enzyme-linked immunosorbent assay

Detailed descriptions of the procedures are not provided here, as they are standard and widely used in the research field. Enzyme-linked immunosorbent assay (ELISA) was performed as previously described (13). For detection of urinary albumin, microtiter plates were coated with 50 μl of anti-mouse albumin Ab (Bethyl Laboratories), diluted to 1 μg/ml with 0.1 M sodium bicarbonate (WAKO), by incubating in room temperature (RT) for 60 min. After washing the plates five times with PBS containing 0.05% Tween 20 (wash solution), they were blocked with 100 μl of PBS containing 3% BSA and 0.05% Tween 20. After washing, the plates were serially incubated with 50 μl of diluted urine samples (1:10,000) or 50 μl of serially diluted biotinylated albumin (1:5000; Bethyl Laboratories) at RT for 60 min, and 50 μl of goat anti-mouse albumin Abs conjugated with horseradish peroxidase (HRP) (1:3000; Abcam) at RT for 60 min. After washing, bound reactants were detected by 1 min incubation with 3,3’, 5,5’-tetramethylbenzidine. Absorbance was determined at 450 nm.

### 2.7 Blood urea nitrogen analysis

Blood urea nitrogen concentration was analyzed using a Fuji-DRY-CHEM 7000V (Fujifilm).

### 2.8 Statistical analyses

Statistical analyses were performed using GraphPad PRISM software, version 9.0 (GraphPad Software, La Jolla, CA). Comparisons between two groups were analyzed by two-tailed unpaired T test (between two group). Differences at P<0.05 were considered significant.

## 3 Results

### 3.1 Anti-CD4 mAbs successfully eliminated CD4^+^ T cells throughout the body in mice

**Figure 2:**
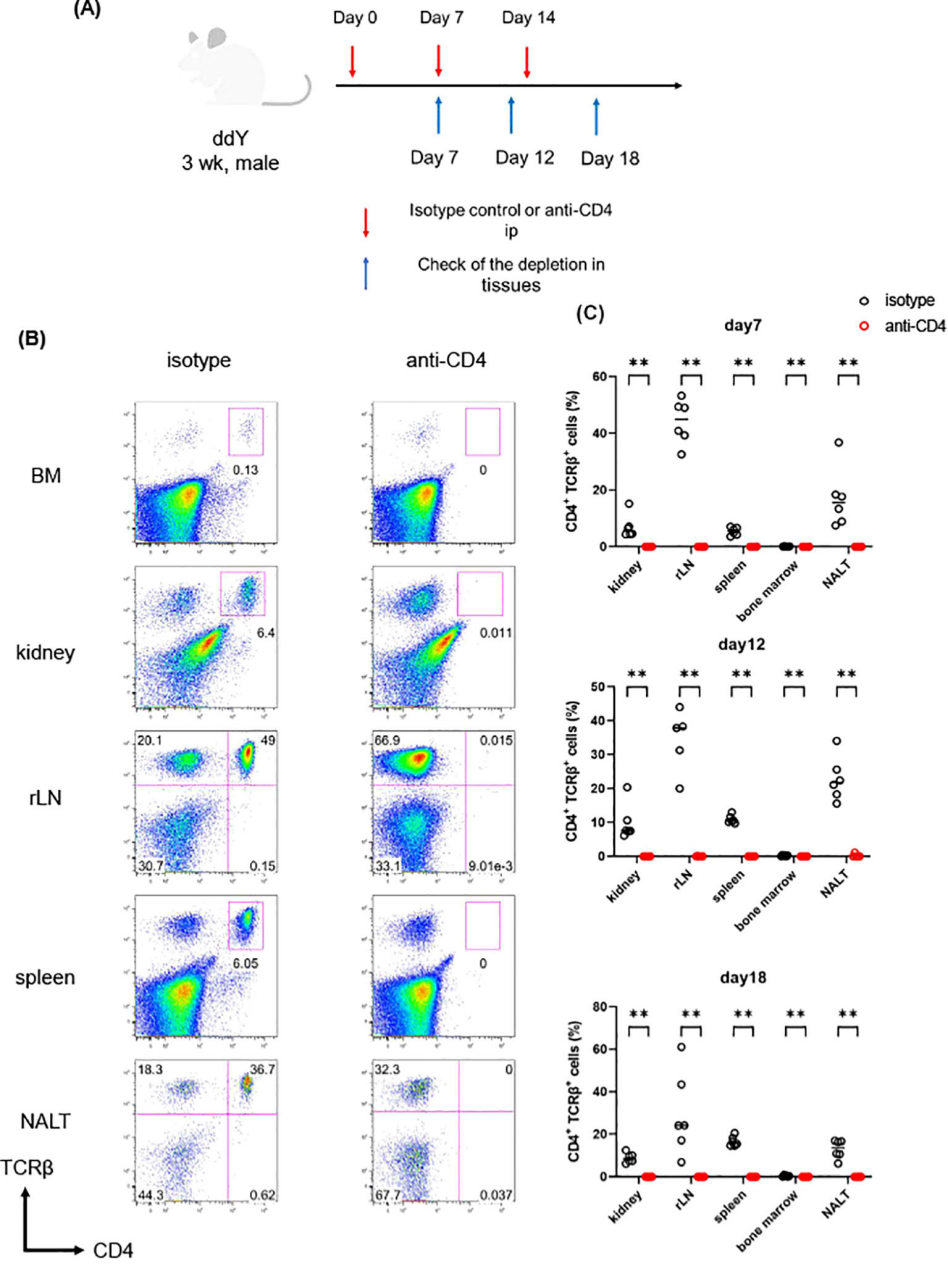
**(A-C)** DdY mice (n=6) were administrated with anti-CD4 or isotype-matched mAbs from 3 wk. At 7, 12 and 18 days after the first administration, leukocytes of kidney, rLN, spleen, BM, NALT were analyzed with flow cytometry. **(A)** The scheme of the experiment of CD4 depletion. **(B)** Leukocytes from tissue of ddY were stained for anti-CD4 and TCRβ mAbs. **(C)** The frequency among the leukocytes of CD4^+^ TCRβ^+^ cells in each mouse tissues. Created with Biorender.com. **p < 0.01.

First, we checked the efficiency of the mAb-mediated depletion of CD4^+^ T cells in lymphoid tissues and kidneys using ddY mice. ddY mice were injected i.p. with anti-CD4 or isotype-matched control mAbs once, twice, or three times, and sacrificed 7, 5, or 4 days after the last injection as shown in **Figure 2**. We analyzed leukocytes in kidney, rLN, spleen, BM and NALT from these mice. As a result, CD4^+^ T cells were almost completely depleted at all organs examined even after a single injection of the anti-CD4 mAb (see **Figures 2B, C**), confirming that administration of mAb systemically depleted CD4^+^ cells in mice.

### 3.2 Depletion of CD4^+^ cells resulted in reduction of IgA^+^ plasmablasts in gddY mouse kidneys

**Figure 3:**
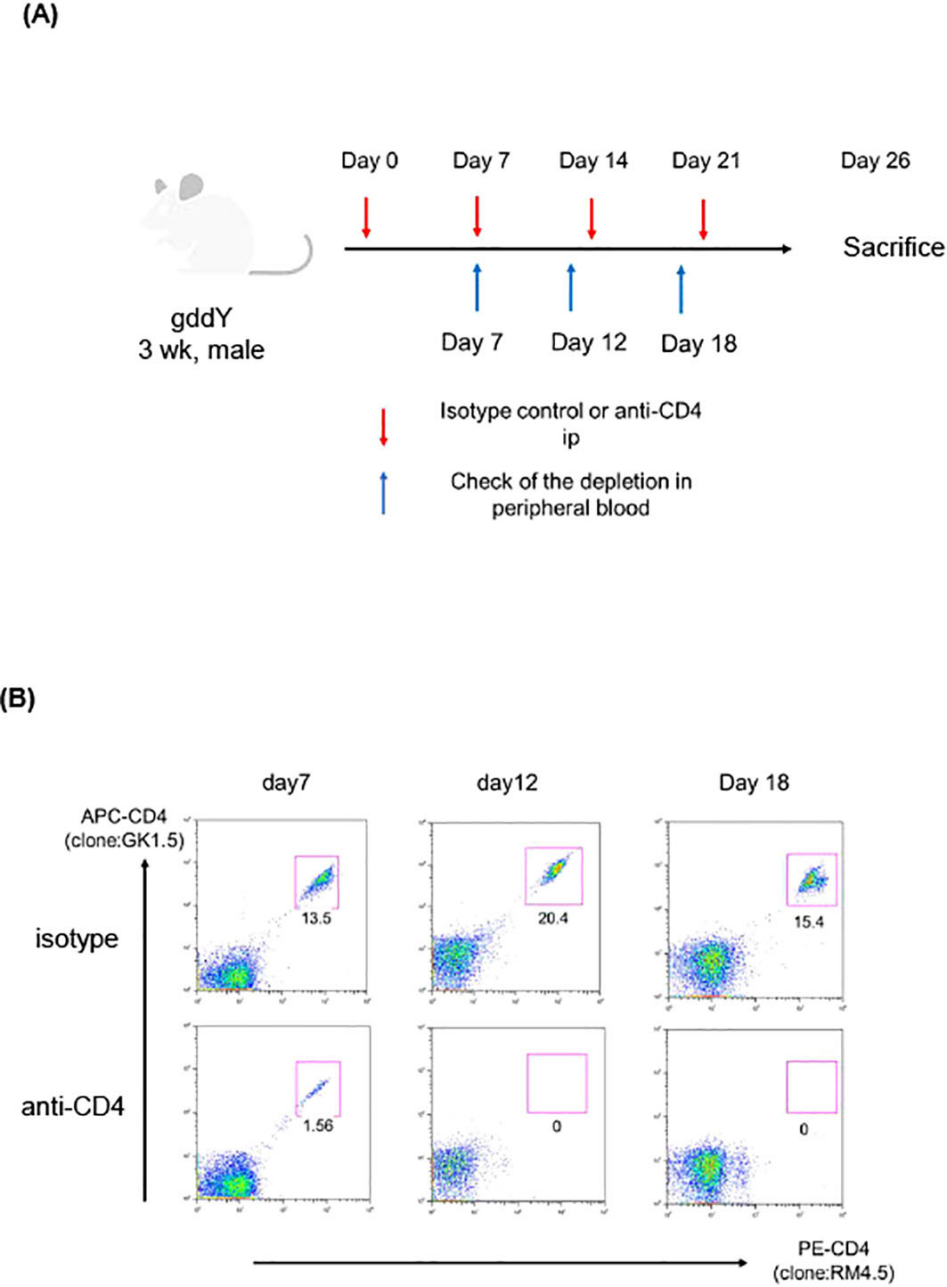
**(A, B)** GddY mice (n=4) were administrated with anti-CD4 or isotype-matched Abs from 3 wk of age for 4 times. At 7, 12, 18 days after the first administration, leukocytes of peripheral blood were analyzed with flow cytometry, and 4 weeks after the first administration, leukocytes isolated from kidneys or rLN were analyzed. **(A)** The scheme of the experiment of CD4 depletion. **(B)** Leukocytes from peripheral blood of gddY were stained for two different anti-CD4 mAbs (clone; GK1.5 or RM4.5). Created with Biorender.com.

**Figure 4:**
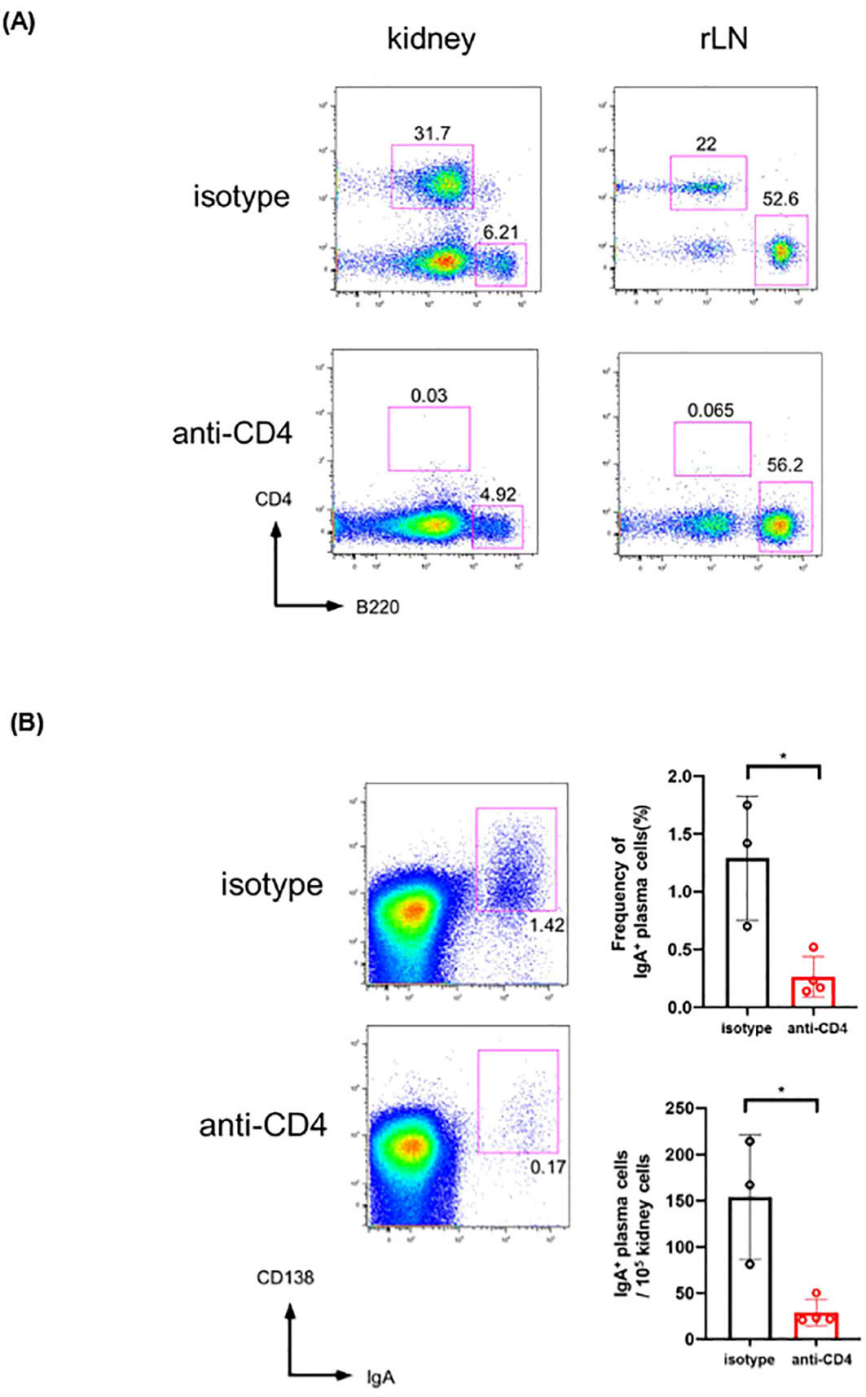
**(A)** Leukocytes isolated from kidneys or rLN were surface stained for CD4 and B220. **(B)** Leukocytes isolated from kidneys were surface stained for CD138 then intracellularly for IgA (left). The frequency among the leukocytes (top) and the number (bottom) of the IgA^+^ CD138^+^ cells in each mouse kidney. *p < 0.05.

Next, we depleted CD4^+^ T cells in gddY by repeatedly administrating anti-CD4 mAb as shown in **Figure 3**. We confirmed that CD4^+^ cells were almost completely depleted in the peripheral blood after the second and third injection, i.e. day 12 and 18 after the primary administration (see **Figure 3B**). Five days after the final (fourth) injection of mAbs (7 weeks of age), these mice were dissected, and lymphocytes isolated from kidneys and rLNs were analyzed. We confirmed that CD4^+^ cells were essentially depleted in the kidney and rLN by the anti-CD4 mAb administration (see **Figure 4A**). In the same mice, we found that the frequency and number of IgA^+^ PBs in the kidneys were significantly reduced by the anti-CD4 treatment (see **Figure 4B**), indicating that CD4^+^ T cells are necessary for generation or maintenance of the PBs in the kidneys of gddY mice.

### 3.3 Depletion of CD4^+^ T cells did not affect pathogenesis in gddY mice

**Figure 5:**
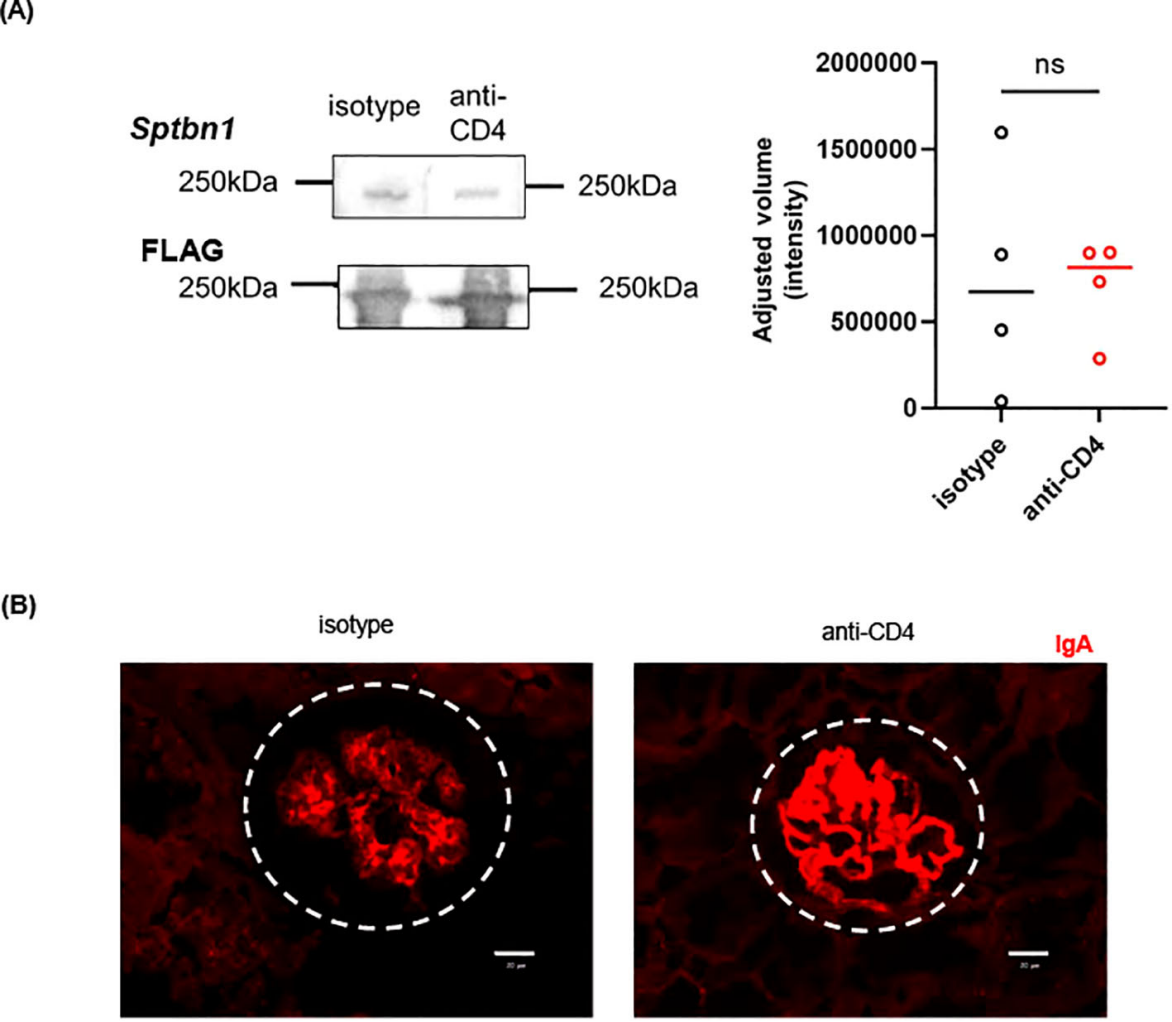
**(A)** Western blotting of recombinant Sptbn1 protein, blotted with sera from 7-wk gddY mice administrated with anti-CD4 or isotype-matched Abs as in **Figure 2** (n=4), followed with anti-IgA Ab. **(B)** Immunofluorescence microscopy of kidney sections of the mice excised at 7 wk in (see **Figure 2A**) were stained with anti-IgA (red). Representative immunofluorescence microscopy images are shown. Dashed circles indicate areas of glomeruli and white lines indicate scale bars (50 μm). ns, not significant.

**Figure 6:**
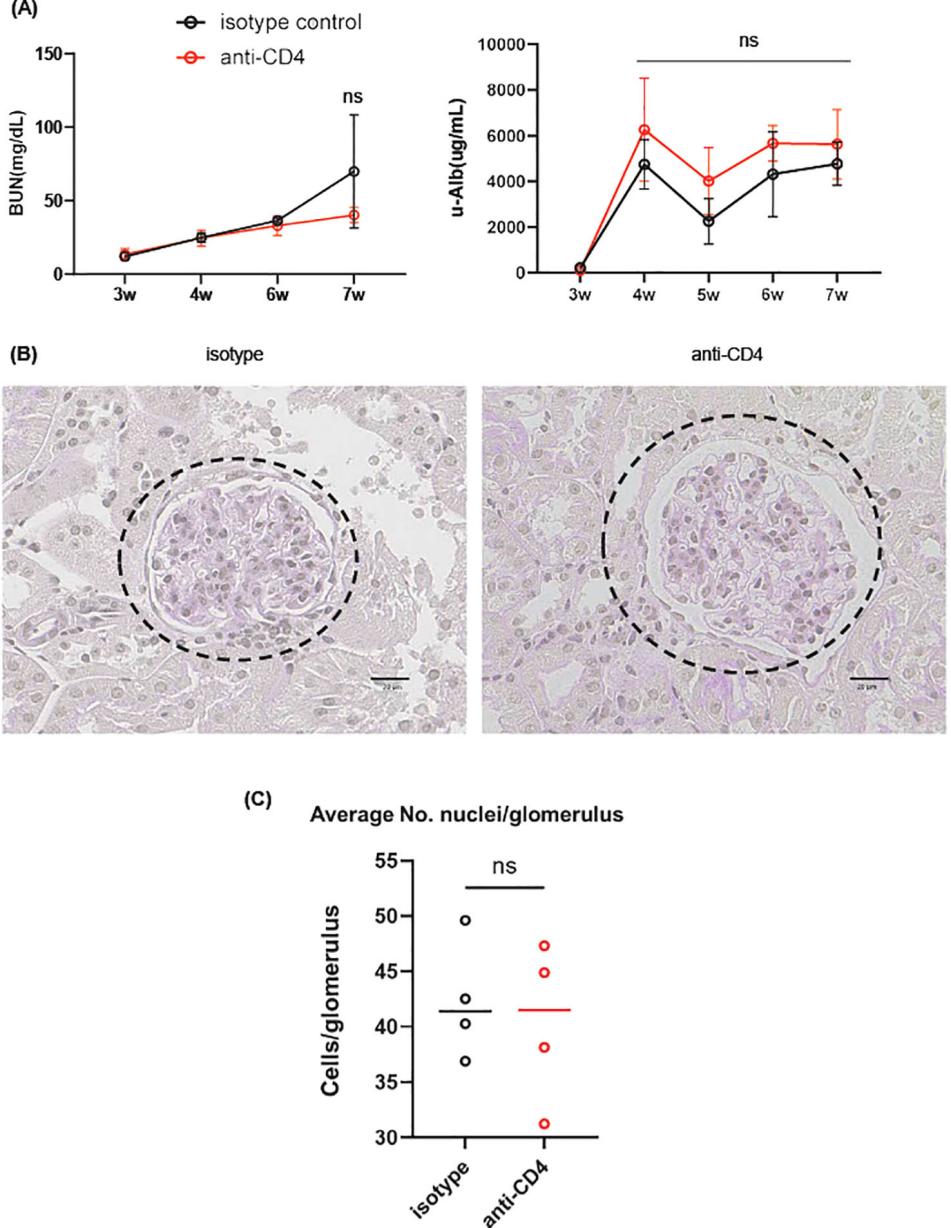
**(A)** Blood urea nitrogen and urinary albumin concentrations of gddY mice, 1, 2, 3 and 4 wk after i.p. administration of isotype-matched or anti-CD4 Abs at 3 wk of age as in **Figure 2** (n=4). **(B, C)** PAS staining of the kidney sections used in **Figure 4**. Dashed circles indicate areas of glomeruli and black lines indicate scale bars (50 μm). The average number of nuclei per glomerulus, counted visually in 20 glomeruli per mouse, is plotted in the bottom panel. ns, not significant.

Given the above results, we examined the effect of the CD4^+^ T cell-depletion on IgAN phenotype of gddY mice. First, we evaluated anti β2-spectrin IgA Abs in the sera of gddY mice treated with anti-CD4 or isotype-matched control Abs with Western blotting. Despite the reduction of IgA^+^ PBs in the kidney, serum anti-β2-spectrin IgA Abs were detected in the anti-CD4-treated mice, to the similar levels as in the control mice (see **Figure 5A**) and glomerular IgA deposition were similar between the two groups (see **Figure 5B**). There was no significant difference in the levels of blood urea nitrogen and urinary albumin (u-Alb) between the two mouse groups at any time points, even after the fourth injection (7w) (see **Figure 6A**). Pathologically, the numbers of the cells in the glomeruli (see **Figures 6B, C**) at 7w were similar between the two groups. These results indicate that depletion of CD4^+^ T cells in gddY mice did not affect the production of serum anti β2-spectrin IgA Abs.

## 4 Discussion

IgAN is the most common glomerulonephritis worldwide and is characterized by the selective deposition of IgA in the glomerular mesangium. Although recent studies have elucidated that autoantibodies targeting mesangial self-antigens contribute to glomerular specificity, the pathogenicity of these autoantibodies has not yet been fully established. Demonstrating their pathogenic roles and clarifying the mechanisms underlying autoantibody production therefore remain urgent issues.

Although the pathogenic involvement of T cells in human IgAN has also been reported (21), in this study, we focused on the dependence of autoantibody production on CD4 T cells. Based on our previous findings, we hypothesized that PBs infiltrating the kidneys of gddY mice—which are generated in a CD4 T cell–dependent manner—may serve as a source of serum anti-β2-spectrin IgA autoantibodies. Our results demonstrated that 1) renal PBs are generated in a CD4 T cell–dependent manner, 2) these PBs do not constitute the major source of serum anti-β2-spectrin IgA autoantibodies, and 3) anti-β2-spectrin IgA autoantibodies are produced independently of CD4 T cells.

The gddY mouse used in the present study closely resembles human IgAN not only phenotypically but also genetically. Importantly, this model has been used in the preclinical evaluation of novel therapeutic agents for IgAN, including drugs currently undergoing clinical trials and those already approved (22, 23). Therefore, the gddY mouse is considered one of the most appropriate models for elucidating the pathophysiology of human IgAN. Indeed, we have accumulated findings using this model that have contributed to a better understanding of the disease mechanisms in humans (24–26). Although validation in human subjects is ultimately required, we believe that the findings obtained in this study provide valuable insights into the pathogenesis of human IgAN.

A previous study using ddY mice, from which gddY mice were derived, reported comparable levels of urinary protein between anti-CD4 mAb–treated and untreated groups (27). However, because ddY mice exhibit marked heterogeneity in the timing of IgAN onset (28), evaluating the effects of CD4 T-cell depletion on disease severity was challenging. To overcome this limitation, we used gddY mice, in which nearly all individuals develop IgAN by 8 weeks of age (10).

Given that renal failure might be reversible (29), early intervention before the onset of renal dysfunction was warranted. In gddY mice, kidney-infiltrating IgA PBs progressively increase with age (13), and renal failure develops by 8 weeks at the latest. Therefore, following a previous report (30), we initiated treatment at 3 weeks of age.

Previous studies have demonstrated that PB generation is CD4 T cell–dependent (31); thus, the observed reduction in PBs following CD4 depletion was expected. Importantly, we confirm here that renal PBs are also generated in a T cell–dependent manner. It has been reported that patients with human IgAN exhibit an increased frequency of IgA^+^ PBs in the peripheral blood, which secrete Gd-IgA1 (32). In systemic lupus erythematosus, particularly lupus nephritis, kidney-resident PCs are known to localize within the kidney and are considered potentially pathogenic (33). The number of PCs infiltrating the kidneys has been reported to correlate with the severity of renal injury in patients with IgAN (13). This observation raises the possibility that PBs may exert pathogenic effects beyond antibody production, potentially through cytokine secretion. However, this hypothesis requires further investigation.

We next assessed disease phenotypes following PB reduction but found no significant improvement in IgAN features after CD4 T-cell depletion, even in gddY mice. This suggests that serum anti-β2-spectrin IgA autoantibodies are produced in a CD4 T cell–independent manner. While PBs are thought to have nephritogenic potential, depletion of these cells did not alter the renal injury phenotype. This finding is consistent with the multi-hit hypothesis (34), which proposes that the primary driver of inflammation in IgAN is the deposition of IgA within the glomeruli, rather than PB-mediated injury alone.

Although depletion of CD4 T cells markedly reduced renal IgA^+^ PBs, serum anti-β2-spectrin IgA titers remained unchanged, indicating that IgA autoantibodies continued to be produced by PBs residing outside the kidneys. Previous studies have suggested that nephritogenic IgA is primarily produced in mucosal tissues and bone marrow in both model animals and patients with IgAN (35). Thus, CD4 T cells might regulate the recruitment of IgA^+^ PBs into the kidney, whereas autoantibody-producing PBs in other organs are unaffected. Comprehensive identification of the tissues responsible for IgA autoantibody production in gddY mice will be an important future task.

The involvement of the gut–kidney axis in IgAN pathogenesis has been well documented (36, 37), and assessing intestinal IgA PBs is indeed relevant. However, we did not evaluate gut PBs in this study. The primary site of pathogenic IgA production—NALT versus GALT—has been debated for decades. A previous study in ddY mice indicated that NALT is the predominant source of aberrantly glycosylated IgA (24). Based on this, we did not prioritize intestinal PBs, although the NALT–GALT debate remains unresolved.

PBs producing IgA with extensive somatic hypermutations might exist in various organs. Our study did not confirm whether such PBs were fully eliminated by anti-CD4 mAb treatment. Nevertheless, it is noteworthy that IgA autoantibodies continued to be produced in a CD4 T cell–independent manner in gddY mice. Several mechanisms might explain this observation. GC-independent class-switch recombination and somatic hypermutation have been reported in liver B cells in response to E. muris infection (38). Additionally, a CD4 T cell–independent but group 2 innate lymphoid cells-dependent IgA response regulated by commensal microbes, including H. pylori, has been described (39). Another possibility is that IgA^+^ PBs or IgA^+^ memory B cells (MBCs) generated before 3 weeks of age continued to produce pathogenic IgA. This is consistent with a recent report showing that antibiotic treatment from 2 weeks—but not 8 weeks—of age reduced IgA autoantibody production and renal PB infiltration in gddY mice, suggesting that commensal bacteria provide sensitizing antigens (30).

Although CD4 T cells play essential roles in activating MBCs under normal conditions, IgAN progressed in the absence of CD4 T cells after 3 weeks of age in our study. This raises the question of how IgA^+^ MBCs are reactivated in a T cell–independent manner (40, 41). TLR7-mediated activation of B cells has been implicated in renal inflammation and Gd-IgA1 synthesis in human IgAN (42), and activation of TLR7 and TLR9 contributes to nephritis in mouse IgAN models (25, 26). Thus, IgA^+^ MBCs might be reactivated through TLR signaling independently of T cells.

Patients with IgAN show increased double-negative (DN) B cells (43), and in systemic lupus erythematosus, expansion of this subset—characterized by high TLR7 expression—is a well-known feature (44). These findings suggest that TLR7-driven activation of DN B cells might also contribute to IgAN. Evaluating TLR7 and MyD88 expression in DN B cells in gddY mice might therefore help elucidate T cell–independent autoantibody production.

Moreover, type-2-polarized MBCs have been shown to maintain IgE memory in allergic patients (45), with IgG1-expressing B cells readily differentiating into IgE^+^ cells. By analogy, IgAN may involve IgA-prone MBCs. Our findings indicate that serum anti-β2-spectrin IgA Auto-Abs are generated in a CD4 T cell–independent manner in gddY mice. We speculate that pathogenic MBCs producing IgA^+^ PBs arise during an early CD4 T cell–dependent sensitization phase but that disease progression subsequently continues independently of CD4 T cells once MBCs have been established. Further investigation is required to elucidate alternative T cell–independent pathways of autoantibody production.

In conclusion, we demonstrate that serum anti-β2-spectrin IgA autoantibodies are produced independently of CD4 T cells in a murine model of IgAN. Although the precise mechanisms and anatomical sources of pathogenic IgA remain unresolved, our findings provide new insight into the pathogenesis of IgAN.

## Data Availability

The original contributions presented in the study are included in the article/supplementary material. Further inquiries can be directed to the corresponding author.
